# Supplementary material for: Utilizing NF-κB Signaling in Porcine Epithelial Cells to Identify a Plant-Based Additive for the Development of a Porcine Epidemic Diarrhea Virus Vaccine
Source: Vet Sci. 2025 Feb 18;12(2):181. doi: 10.3390/vetsci12020181 (PMC11860592; doi:10.3390/vetsci12020181)
Supplement: Supplementary file 1 [file vetsci-12-00181-s001.zip › Supplementary File S1.pdf]

Supplementary File S1

Supplemental data for Figure 3

Panel A

ANOVA - TNF-α (ng/mL)

								95% CI for ω²	
Homogeneity Correction	Cases	Sum of Squares	df	Mean Square	F	p	ω²	Lower	Upper
None	Concentration	81.490	5.000	16.298	216.920	< .001	0.984	0.955	0.992
	Residuals	0.902	12.000	0.075					
Welch	Concentration	81.490	5.000	16.298	333.648	< .001	0.984	0.955	0.992
	Residuals	0.902	5.419	0.166					

Note. Type III Sum of Squares

Kruskal-Wallis Test

Kruskal-Wallis Test

					95% CI for Rank ε²	
Factor	Statistic	df	p	Rank ε²	Lower	Upper
Concentration	16.579	5	0.005	0.975	0.978	0.990

Dunn

Dunn's Post Hoc Comparisons - Concentration

Comparison	z	W_i	W_j	r_{rb}	p	p_{bonf}	p_{holm}
0 - 0.63	-0.688	2.000	5.000	1.000	0.491	1.000	1.000
0 - 1.25	-1.376	2.000	8.000	1.000	0.169	1.000	1.000
0 - 2.5	-2.065	2.000	11.000	1.000	0.039	0.584	0.467
0 - 5	-2.753	2.000	14.000	1.000	0.006	0.089	0.083
0 - 10	-3.441	2.000	17.000	1.000	< .001	0.009	0.009
0.63 - 1.25	-0.688	5.000	8.000	1.000	0.491	1.000	1.000
0.63 - 2.5	-1.376	5.000	11.000	1.000	0.169	1.000	1.000
0.63 - 5	-2.065	5.000	14.000	1.000	0.039	0.584	0.467
0.63 - 10	-2.753	5.000	17.000	1.000	0.006	0.089	0.083
1.25 - 2.5	-0.688	8.000	11.000	1.000	0.491	1.000	1.000
1.25 - 5	-1.376	8.000	14.000	1.000	0.169	1.000	1.000
1.25 - 10	-2.065	8.000	17.000	1.000	0.039	0.584	0.467
2.5 - 5	-0.688	11.000	14.000	1.000	0.491	1.000	1.000
2.5 - 10	-1.376	11.000	17.000	1.000	0.169	1.000	1.000
5 - 10	-0.688	14.000	17.000	1.000	0.491	1.000	1.000

Note. Rank-biserial correlation based on individual Mann-Whitney tests.

Panel B

ANOVA – TNF-α + DMSO

Homogeneity Correction	Cases	Sum of Squares	df	Mean Square	F	p	ω²	95% CI for ω²	
								Lower	Upper
None	Concentration	16.492	5.000	3.298	37.999	< .001	0.911	0.747	0.954
	Residuals	1.042	12.000	0.087					
Welch	Concentration	16.492	5.000	3.298	35.787	< .001	0.911	0.747	0.954
	Residuals	1.042	5.377	0.194					

Note. Type III Sum of Squares

Kruskal-Wallis Test

Kruskal-Wallis Test

Factor	Statistic	df	p	Rank ε²	95% CI for Rank ε²	
					Lower	Upper
Concentration	15.361	5	0.009	0.904	0.892	0.983

Dunn

Dunn's Post Hoc Comparisons - Concentration

Comparison	z	W_i	W_j	r_{rb}	p	p_{bonf}	p_{holm}
0 - 0.63	-0.536	2.500	4.833	0.667	0.592	1.000	1.000
0 - 1.25	-1.186	2.500	7.667	1.000	0.236	1.000	1.000
0 - 2.5	-2.410	2.500	13.000	1.000	0.016	0.239	0.207
0 - 5	-2.257	2.500	12.333	1.000	0.024	0.360	0.288
0 - 10	-3.252	2.500	16.667	1.000	0.001	0.017	0.017
0.63 - 1.25	-0.650	4.833	7.667	0.778	0.515	1.000	1.000
0.63 - 2.5	-1.875	4.833	13.000	1.000	0.061	0.913	0.609
0.63 - 5	-1.722	4.833	12.333	1.000	0.085	1.000	0.766
0.63 - 10	-2.716	4.833	16.667	1.000	0.007	0.099	0.092
1.25 - 2.5	-1.224	7.667	13.000	1.000	0.221	1.000	1.000
1.25 - 5	-1.071	7.667	12.333	1.000	0.284	1.000	1.000
1.25 - 10	-2.066	7.667	16.667	1.000	0.039	0.583	0.427
2.5 - 5	0.153	13.000	12.333	0.111	0.878	1.000	1.000
2.5 - 10	-0.842	13.000	16.667	0.778	0.400	1.000	1.000
5 - 10	-0.995	12.333	16.667	1.000	0.320	1.000	1.000

Note. Rank-biserial correlation based on individual Mann-Whitney tests.

Panel C

Independent Samples T-Test

	Test	Statistic	df	p	Mean Difference	SE Difference	95% CI for Mean Difference		Cohen's d	SE Cohen's d
							Lower	Upper		
Fold-change	Student	-36.653	20.000	< .001	-6.900	0.188	-∞	-6.575	-15.629	3.359
	Welch	-36.653	10.468	< .001	-6.900	0.188	-∞	-6.560	-15.629	3.359

Note. For all tests, the alternative hypothesis specifies that group *Solvent control* is less than group *TNFa (10 ng/mL)* .
