# Supplementary material for: Utilizing NF-κB Signaling in Porcine Epithelial Cells to Identify a Plant-Based Additive for the Development of a Porcine Epidemic Diarrhea Virus Vaccine
Source: Vet Sci. 2025 Feb 18;12(2):181. doi: 10.3390/vetsci12020181 (PMC11860592; doi:10.3390/vetsci12020181)
Supplement: Supplementary file 1 [file vetsci-12-00181-s001.zip › Supplementary File S2.pdf]

Supplementary File S2

Supplemental data Figure 5

ANOVA - Percentage (%)

Cases	Sum of Squares	df	Mean Square	F	p	ω²	95% CI for ω²	
							Lower	Upper
Concentration	1692.363	3	564.121	9.676	< .001	0.416	0.072	0.615
Group	71.363	2	35.681	0.612	0.550	0.000	0.000	0.000
Concentration ✻ Group	426.739	6	71.123	1.220	0.331	0.021	0.000	0.000
Residuals	1399.214	24	58.301					

Note. Type III Sum of Squares

Chamomile

ANOVA - Percentage (%)

Homogeneity Correction	Cases	Sum of Squares	df	Mean Square	F	p
None	Concentration	465.487	3.000	155.162	2.285	0.156
	Residuals	543.341	8.000	67.918		
Welch	Concentration	465.487	3.000	155.162	4.284	0.109
	Residuals	543.341	3.594	151.197		

Note. Type III Sum of Squares

Kruskal-Wallis Test

Kruskal-Wallis Test

Factor	Statistic	df	p	Rank ε²	95% CI for Rank ε²	
					Lower	Upper
Concentration	5.462	3	0.141	0.497	0.306	0.901

Dunn

Dunn's Post Hoc Comparisons - Concentration

Comparison	z	W_i	W_j	r_{rb}	p	p_{bonf}	p_{holm}
0 - 10	0.340	4.667	3.667	0.111	0.734	1.000	1.000
0 - 100	-1.132	4.667	8.000	0.556	0.258	1.000	0.773
0 - 1000	-1.698	4.667	9.667	0.778	0.089	0.537	0.447
10 - 100	-1.472	3.667	8.000	0.778	0.141	0.846	0.564
10 - 1000	-2.038	3.667	9.667	1.000	0.042	0.249	0.249
100 - 1000	-0.566	8.000	9.667	0.333	0.571	1.000	1.000

Note. Rank-biserial correlation based on individual Mann-Whitney tests.

Boerhaavia diffusa

ANOVA - Percentage (%)

Homogeneity Correction	Cases	Sum of Squares	df	Mean Square	F	p	ω²	95% CI for ω²	
								Lower	Upper
None	Concentration	593.116	3.000	197.705	6.391	0.016	0.574	0.000	0.793
	Residuals	247.481	8.000	30.935					
Welch	Concentration	593.116	3.000	197.705	36.563	0.002	0.574	0.000	0.793
	Residuals	247.481	4.241	58.359					

Note. Type III Sum of Squares

Kruskal-Wallis Test

Kruskal-Wallis Test

Factor	Statistic	df	p	Rank ε²	95% CI for Rank ε²	
					Lower	Upper
Concentration	7.333	3	0.062	0.667	0.647	0.968

Dunn

Dunn's Post Hoc Comparisons - Concentration

Comparison	z	W_i	W_j	r_{rb}	p	p_{bonf}	p_{holm}
0 - 10	-0.114	5.667	6.000	0.111	0.909	1.000	1.000
0 - 100	0.800	5.667	3.333	0.333	0.424	1.000	1.000
0 - 1000	-1.828	5.667	11.000	1.000	0.068	0.406	0.338
10 - 100	0.914	6.000	3.333	0.778	0.361	1.000	1.000
10 - 1000	-1.713	6.000	11.000	1.000	0.087	0.520	0.347
100 - 1000	-2.627	3.333	11.000	1.000	0.009	0.052	0.052

Note. Rank-biserial correlation based on individual Mann-Whitney tests.

Mulberry

ANOVA - Percentage (%)

Homogeneity Correction	Cases	Sum of Squares	df	Mean Square	F	p
None	Concentration	1094.992	3.000	364.997	4.734	0.035
	Residuals	616.817	8.000	77.102		
Welch	Concentration	1094.992	3.000	364.997	3.841	0.103
	Residuals	616.817	4.419	139.580		

Note. Type III Sum of Squares

Kruskal-Wallis Test

Kruskal-Wallis Test

					95% CI for Rank ε²	
Factor	Statistic	df	p	Rank ε²	Lower	Upper
Concentration	6.806	3	0.078	0.619	0.583	0.931

Dunn

Dunn's Post Hoc Comparisons - Concentration

Comparison	z	W_i	W_j	r_{rb}	p	p_{bonf}	p_{holm}
0 - 10	0.454	5.167	3.833	0.222	0.650	1.000	1.000
0 - 100	-0.284	5.167	6.000	0.111	0.777	1.000	1.000
0 - 1000	-1.985	5.167	11.000	1.000	0.047	0.283	0.236
10 - 100	-0.737	3.833	6.000	0.556	0.461	1.000	1.000
10 - 1000	-2.439	3.833	11.000	1.000	0.015	0.088	0.088
100 - 1000	-1.701	6.000	11.000	1.000	0.089	0.533	0.355

Note. Rank-biserial correlation based on individual Mann-Whitney tests.

H_2O_2 vs DMSO

Independent Samples T-Test

									95% CI for Cohen's d	
	Test	Statistic	df	p	Mean Difference	SE Difference	Cohen's d	SE Cohen's d	Lower	Upper
Percentage (%)	Student	-67.483	4.000	< .001	-106.689	1.581	-55.099	22.509	-89.870	-19.131
	Welch	-67.483	2.380	< .001	-106.689	1.581	-55.099	22.509	-98.836	-11.231
