# Supplementary material for: Utilizing NF-κB Signaling in Porcine Epithelial Cells to Identify a Plant-Based Additive for the Development of a Porcine Epidemic Diarrhea Virus Vaccine
Source: Vet Sci. 2025 Feb 18;12(2):181. doi: 10.3390/vetsci12020181 (PMC11860592; doi:10.3390/vetsci12020181)
Supplement: Supplementary file 1 [file vetsci-12-00181-s001.zip › Supplementary File S3.pdf]

Supplementary File S3

Supplemental data Figure 7

Repeated Measures ANOVA

Within Subjects Effects

Cases	Sum of Squares	df	Mean Square	F	p	ω²
Day	2084.571[a]	4[a]	521.143[a]	213.750[a]	< .001[a]	0.913
Day ∗ Group	650.895[a]	24[a]	27.121[a]	11.124[a]	< .001[a]	0.389
Residuals	136.533	56	2.438			

Note. Type III Sum of Squares
[a] Mauchly's test of sphericity indicates that the assumption of sphericity is violated (p < .05).

Between Subjects Effects

Cases	Sum of Squares	df	Mean Square	F	p	ω²
Group	959.390	6	159.898	45.872	< .001	0.719
Residuals	48.800	14	3.486			

Note. Type III Sum of Squares

Descriptives

Descriptives

Day	Group	N	Mean	SD	SE	Coefficient of variation
Day 0	IM	3	5.000	0.000	0.000	0.000
	IM 120	3	5.000	0.000	0.000	0.000
	IM 480	3	5.000	0.000	0.000	0.000
	OR	3	5.000	0.000	0.000	0.000
	OR 120	3	5.000	0.000	0.000	0.000
	OR 480	3	5.000	0.000	0.000	0.000
	PBS	3	5.000	0.000	0.000	0.000
Day 7	IM	3	7.000	0.000	0.000	0.000
	IM 120	3	7.667	2.309	1.333	0.301
	IM 480	3	8.333	1.155	0.667	0.139
	OR	3	5.000	0.000	0.000	0.000
	OR 120	3	5.000	0.000	0.000	0.000
	OR 480	3	5.000	0.000	0.000	0.000
	PBS	3	5.000	0.000	0.000	0.000
Day 14	IM	3	15.667	2.309	1.333	0.147
	IM 120	3	15.000	1.732	1.000	0.115
	IM 480	3	15.667	2.309	1.333	0.147
	OR	3	9.667	2.309	1.333	0.239
	OR 120	3	15.000	1.732	1.000	0.115
	OR 480	3	13.667	2.309	1.333	0.169
	PBS	3	5.000	0.000	0.000	0.000
Day 21	IM	3	19.667	0.577	0.333	0.029
	IM 120	3	19.000	0.000	0.000	0.000
	IM 480	3	19.667	0.577	0.333	0.029
	OR	3	11.667	0.577	0.333	0.049
	OR 120	3	7.000	6.928	4.000	0.990
	OR 480	3	13.667	0.577	0.333	0.042
	PBS	3	5.000	0.000	0.000	0.000
Day 28	IM	3	21.667	0.577	0.333	0.027
	IM 120	3	20.333	1.155	0.667	0.057
	IM 480	3	21.000	1.732	1.000	0.082

Descriptives

Day	Group	N	Mean	SD	SE	Coefficient of variation
	OR	3	15.000	1.732	1.000	0.115
	OR 120	3	14.333	1.155	0.667	0.081
	OR 480	3	17.667	0.577	0.333	0.033
	PBS	3	5.000	0.000	0.000	0.000

Post Hoc Tests

Post Hoc Comparisons - Group ✻ Day - Conditional on Group

				95% CI for Mean Difference						95% CI for Cohen's d		
Group			Mean Difference	Lower	Upper	SE	df	t	Cohen's d	Lower	Upper	p_{bonf}
IM	Day 0	Day 7	-2.000	-3.874	-0.126	0.563	14	-3.550	-1.229	-3.506	1.048	0.032
		Day 14	-10.667	-14.461	-6.872	1.141	14	-9.350	-6.555	-14.328	1.217	< .001
		Day 21	-14.667	-19.764	-9.569	1.533	14	-9.569	-9.014	-19.642	1.614	< .001
		Day 28	-16.667	-18.884	-14.450	0.667	14	-25.000	-10.243	-21.046	0.560	< .001
	Day 7	Day 14	-8.667	-11.966	-5.367	0.992	14	-8.736	-5.326	-11.752	1.099	< .001
		Day 21	-12.667	-18.032	-7.301	1.613	14	-7.851	-7.785	-17.471	1.902	< .001
		Day 28	-14.667	-16.540	-12.793	0.563	14	-26.031	-9.014	-18.505	0.477	< .001
	Day 14	Day 21	-4.000	-10.885	2.885	2.070	14	-1.932	-2.458	-9.854	4.937	0.738
		Day 28	-6.000	-7.965	-4.035	0.591	14	-10.153	-3.687	-7.978	0.603	< .001
	Day 21	Day 28	-2.000	-8.043	4.043	1.817	14	-1.101	-1.229	-7.457	4.999	1.000
IM 120	Day 0	Day 7	-2.667	-4.540	-0.793	0.563	14	-4.733	-1.639	-4.175	0.898	0.003
		Day 14	-10.000	-13.794	-6.206	1.141	14	-8.765	-6.146	-13.553	1.262	< .001
		Day 21	-14.000	-19.097	-8.903	1.533	14	-9.134	-8.604	-18.864	1.656	< .001
		Day 28	-15.333	-17.550	-13.116	0.667	14	-23.000	-9.423	-19.401	0.554	< .001
	Day 7	Day 14	-7.333	-10.633	-4.034	0.992	14	-7.392	-4.507	-10.226	1.212	< .001
		Day 21	-11.333	-16.699	-5.968	1.613	14	-7.024	-6.965	-15.963	2.033	< .001
		Day 28	-12.667	-14.540	-10.793	0.563	14	-22.481	-7.785	-16.037	0.467	< .001
	Day 14	Day 21	-4.000	-10.885	2.885	2.070	14	-1.932	-2.458	-9.854	4.937	0.738
		Day 28	-5.333	-7.299	-3.368	0.591	14	-9.025	-3.278	-7.198	0.643	< .001
	Day 21	Day 28	-1.333	-7.376	4.710	1.817	14	-0.734	-0.819	-6.975	5.336	1.000
IM 480	Day 0	Day 7	-3.333	-5.207	-1.460	0.563	14	-5.916	-2.049	-4.885	0.787	< .001
		Day 14	-10.667	-14.461	-6.872	1.141	14	-9.350	-6.555	-14.328	1.217	< .001
		Day 21	-14.667	-19.764	-9.569	1.533	14	-9.569	-9.014	-19.642	1.614	< .001
		Day 28	-16.000	-18.217	-13.783	0.667	14	-24.000	-9.833	-20.223	0.557	< .001
	Day 7	Day 14	-7.333	-10.633	-4.034	0.992	14	-7.392	-4.507	-10.226	1.212	< .001
		Day 21	-11.333	-16.699	-5.968	1.613	14	-7.024	-6.965	-15.963	2.033	< .001
		Day 28	-12.667	-14.540	-10.793	0.563	14	-22.481	-7.785	-16.037	0.467	< .001
	Day 14	Day 21	-4.000	-10.885	2.885	2.070	14	-1.932	-2.458	-9.854	4.937	0.738
		Day 28	-5.333	-7.299	-3.368	0.591	14	-9.025	-3.278	-7.198	0.643	< .001
	Day 21	Day 28	-1.333	-7.376	4.710	1.817	14	-0.734	-0.819	-6.975	5.336	1.000
OR	Day 0	Day 7	-1.389×10^{-15}	-1.874	1.874	0.563	14	-2.465×10^{-15}	-1.776×10^{-15}	-1.891	1.891	1.000
		Day 14	-4.667	-8.461	-0.872	1.141	14	-4.090	-2.868	-7.707	1.971	0.011
		Day 21	-6.667	-11.764	-1.569	1.533	14	-4.350	-4.097	-10.755	2.561	0.007
		Day 28	-10.000	-12.217	-7.783	0.667	14	-15.000	-6.146	-12.870	0.579	< .001
	Day 7	Day 14	-4.667	-7.966	-1.367	0.992	14	-4.704	-2.868	-7.322	1.586	0.003
		Day 21	-6.667	-12.032	-1.301	1.613	14	-4.132	-4.097	-10.966	2.772	0.010
		Day 28	-10.000	-11.874	-8.126	0.563	14	-17.748	-6.146	-12.763	0.472	< .001
	Day 14	Day 21	-2.000	-8.885	4.885	2.070	14	-0.966	-1.229	-8.291	5.832	1.000

Post Hoc Comparisons - Group ✻ Day - Conditional on Group

Group			Mean Difference	95% CI for Mean Difference Lower	95% CI for Mean Difference Upper	SE	df	t	Cohen's d	95% CI for Cohen's d Lower	95% CI for Cohen's d Upper	p_{bonf}
		Day 28	-5.333	-7.299	-3.368	0.591	14	-9.025	-3.278	-7.198	0.643	< .001
	Day 21	Day 28	-3.333	-9.376	2.710	1.817	14	-1.834	-2.049	-8.502	4.405	0.879
OR 120	Day 0	Day 7	-1.611×10^{-15}	-1.874	1.874	0.563	14	-2.859×10^{-15}	-1.776×10^{-15}	-1.891	1.891	1.000
		Day 14	-10.000	-13.794	-6.206	1.141	14	-8.765	-6.146	-13.553	1.262	< .001
		Day 21	-2.000	-7.097	3.097	1.533	14	-1.305	-1.229	-6.526	4.068	1.000
		Day 28	-9.333	-11.550	-7.116	0.667	14	-14.000	-5.736	-12.063	0.591	< .001
	Day 7	Day 14	-10.000	-13.299	-6.701	0.992	14	-10.080	-6.146	-13.308	1.016	< .001
		Day 21	-2.000	-7.366	3.366	1.613	14	-1.240	-1.229	-6.790	4.331	1.000
		Day 28	-9.333	-11.207	-7.460	0.563	14	-16.565	-5.736	-11.949	0.477	< .001
	Day 14	Day 21	8.000	1.115	14.885	2.070	14	3.864	4.917	-3.685	13.519	0.017
		Day 28	0.667	-1.299	2.632	0.591	14	1.128	0.410	-1.618	2.437	1.000
	Day 21	Day 28	-7.333	-13.376	-1.290	1.817	14	-4.036	-4.507	-12.175	3.161	0.012
OR 480	Day 0	Day 7	-7.225×10^{-16}	-1.874	1.874	0.563	14	-1.282×10^{-15}	-8.882×10^{-16}	-1.891	1.891	1.000
		Day 14	-8.667	-12.461	-4.872	1.141	14	-7.597	-5.326	-12.024	1.372	< .001
		Day 21	-8.667	-13.764	-3.569	1.533	14	-5.654	-5.326	-12.853	2.201	< .001
		Day 28	-12.667	-14.884	-10.450	0.667	14	-19.000	-7.785	-16.123	0.554	< .001
	Day 7	Day 14	-8.667	-11.966	-5.367	0.992	14	-8.736	-5.326	-11.752	1.099	< .001
		Day 21	-8.667	-14.032	-3.301	1.613	14	-5.372	-5.326	-13.041	2.388	< .001
		Day 28	-12.667	-14.540	-10.793	0.563	14	-22.481	-7.785	-16.037	0.467	< .001
	Day 14	Day 21	-3.543×10^{-15}	-6.885	6.885	2.070	14	-1.711×10^{-15}	-3.553×10^{-15}	-6.947	6.947	1.000
		Day 28	-4.000	-5.965	-2.035	0.591	14	-6.769	-2.458	-5.678	0.761	< .001
	Day 21	Day 28	-4.000	-10.043	2.043	1.817	14	-2.201	-2.458	-9.062	4.145	0.450
PBS	Day 0	Day 7	-5.879×10^{-15}	-1.874	1.874	0.563	14	-1.043×10^{-14}	-3.553×10^{-15}	-1.891	1.891	1.000
		Day 14	-1.221×10^{-14}	-3.794	3.794	1.141	14	-1.070×10^{-14}	-7.550×10^{-15}	-3.828	3.828	1.000
		Day 21	-1.932×10^{-14}	-5.097	5.097	1.533	14	-1.261×10^{-14}	-1.155×10^{-14}	-5.143	5.143	1.000
		Day 28	-4.991×10^{-15}	-2.217	2.217	0.667	14	-7.486×10^{-15}	-3.109×10^{-15}	-2.237	2.237	1.000
	Day 7	Day 14	-6.328×10^{-15}	-3.299	3.299	0.992	14	-6.379×10^{-15}	-3.997×10^{-15}	-3.329	3.329	1.000
		Day 21	-1.344×10^{-14}	-5.366	5.366	1.613	14	-8.332×10^{-15}	-7.994×10^{-15}	-5.414	5.414	1.000
		Day 28	8.882×10^{-16}	-1.874	1.874	0.563	14	1.576×10^{-15}	4.441×10^{-16}	-1.891	1.891	1.000
	Day 14	Day 21	-7.115×10^{-15}	-6.885	6.885	2.070	14	-3.437×10^{-15}	-3.997×10^{-15}	-6.947	6.947	1.000
		Day 28	7.216×10^{-15}	-1.965	1.965	0.591	14	1.221×10^{-14}	4.441×10^{-15}	-1.983	1.983	1.000
	Day 21	Day 28	1.433×10^{-14}	-6.043	6.043	1.817	14	7.887×10^{-15}	8.438×10^{-15}	-6.097	6.097	1.000

Note. P-value and confidence intervals adjusted for comparing a family of 10 estimates (confidence intervals corrected using the bonferroni method).

Post Hoc Comparisons - Group ✻ Day - Conditional on Day

Day			Mean Difference	95% CI for Mean Difference Lower	95% CI for Mean Difference Upper	SE	df	t	Cohen's d	95% CI for Cohen's d Lower	95% CI for Cohen's d Upper	p_{bonf}
Day 0	IM	IM, 120	1.085×10^{-30}	-5.365×10^{-15}	5.365×10^{-15}	1.450×10^{-15}	14	7.479×10^{-16}	0.000	0.000	0.000	1.000
		IM, 480	-5.423×10^{-31}	-5.365×10^{-15}	5.365×10^{-15}	1.450×10^{-15}	14	-3.739×10^{-16}	0.000	0.000	0.000	1.000
		OR	-1.085×10^{-30}	-5.365×10^{-15}	5.365×10^{-15}	1.450×10^{-15}	14	-7.479×10^{-16}	0.000	0.000	0.000	1.000
		OR, 120	-8.875×10^{-31}	-5.365×10^{-15}	5.365×10^{-15}	1.450×10^{-15}	14	-6.119×10^{-16}	0.000	0.000	0.000	1.000
		OR, 480	-8.875×10^{-31}	-5.365×10^{-15}	5.365×10^{-15}	1.450×10^{-15}	14	-6.119×10^{-16}	0.000	0.000	0.000	1.000

Post Hoc Comparisons - Group ✻ Day - Conditional on Day

Day			Mean Difference	95% CI for Mean Difference Lower	95% CI for Mean Difference Upper	SE	df	t	Cohen's d	95% CI for Cohen's d Lower	95% CI for Cohen's d Upper	p_{bonf}
		PBS	2.713×10^{-15}	-2.652×10^{-15}	8.079×10^{-15}	1.450×10^{-15}	14	1.871	1.332×10^{-15}	-4.068×10^{-8}	4.068×10^{-8}	1.000
	IM, 120	IM, 480	-1.627×10^{-30}	-5.365×10^{-15}	5.365×10^{-15}	1.450×10^{-15}	14	-1.122×10^{-15}	0.000	0.000	0.000	1.000
		OR	-2.169×10^{-30}	-5.365×10^{-15}	5.365×10^{-15}	1.450×10^{-15}	14	-1.496×10^{-15}	0.000	0.000	0.000	1.000
		OR, 120	-1.972×10^{-30}	-5.365×10^{-15}	5.365×10^{-15}	1.450×10^{-15}	14	-1.360×10^{-15}	0.000	0.000	0.000	1.000
		OR, 480	-1.972×10^{-30}	-5.365×10^{-15}	5.365×10^{-15}	1.450×10^{-15}	14	-1.360×10^{-15}	0.000	0.000	0.000	1.000
		PBS	2.713×10^{-15}	-2.652×10^{-15}	8.079×10^{-15}	1.450×10^{-15}	14	1.871	1.332×10^{-15}	-4.068×10^{-8}	4.068×10^{-8}	1.000
	IM, 480	OR	-5.423×10^{-31}	-5.365×10^{-15}	5.365×10^{-15}	1.450×10^{-15}	14	-3.739×10^{-16}	0.000	0.000	0.000	1.000
		OR, 120	-3.451×10^{-31}	-5.365×10^{-15}	5.365×10^{-15}	1.450×10^{-15}	14	-2.380×10^{-16}	0.000	0.000	0.000	1.000
		OR, 480	-3.451×10^{-31}	-5.365×10^{-15}	5.365×10^{-15}	1.450×10^{-15}	14	-2.380×10^{-16}	0.000	0.000	0.000	1.000
		PBS	2.713×10^{-15}	-2.652×10^{-15}	8.079×10^{-15}	1.450×10^{-15}	14	1.871	1.332×10^{-15}	-4.068×10^{-8}	4.068×10^{-8}	1.000
	OR	OR, 120	1.972×10^{-31}	-5.365×10^{-15}	5.365×10^{-15}	1.450×10^{-15}	14	1.360×10^{-16}	0.000	0.000	0.000	1.000
		OR, 480	1.972×10^{-31}	-5.365×10^{-15}	5.365×10^{-15}	1.450×10^{-15}	14	1.360×10^{-16}	0.000	0.000	0.000	1.000
		PBS	2.713×10^{-15}	-2.652×10^{-15}	8.079×10^{-15}	1.450×10^{-15}	14	1.871	1.332×10^{-15}	-4.068×10^{-8}	4.068×10^{-8}	1.000
	OR, 120	OR, 480	0.000	-5.365×10^{-15}	5.365×10^{-15}	1.450×10^{-15}	14	0.000	0.000	0.000	0.000	1.000
		PBS	2.713×10^{-15}	-2.652×10^{-15}	8.079×10^{-15}	1.450×10^{-15}	14	1.871	1.332×10^{-15}	-4.068×10^{-8}	4.068×10^{-8}	1.000
	OR, 480	PBS	2.713×10^{-15}	-2.652×10^{-15}	8.079×10^{-15}	1.450×10^{-15}	14	1.871	1.332×10^{-15}	-4.068×10^{-8}	4.068×10^{-8}	1.000
Day 7	IM	IM, 120	-0.667	-3.614	2.281	0.797	14	-0.837	-0.410	-3.117	2.297	1.000
		IM, 480	-1.333	-4.281	1.614	0.797	14	-1.673	-0.819	-3.624	1.985	1.000
		OR	2.000	-0.948	4.948	0.797	14	2.510	1.229	-1.730	4.188	0.525
		OR, 120	2.000	-0.948	4.948	0.797	14	2.510	1.229	-1.730	4.188	0.525
		OR, 480	2.000	-0.948	4.948	0.797	14	2.510	1.229	-1.730	4.188	0.525
		PBS	2.000	-0.948	4.948	0.797	14	2.510	1.229	-1.730	4.188	0.525
	IM, 120	IM, 480	-0.667	-3.614	2.281	0.797	14	-0.837	-0.410	-3.117	2.297	1.000
		OR	2.667	-0.281	5.614	0.797	14	3.347	1.639	-1.525	4.803	0.101
		OR, 120	2.667	-0.281	5.614	0.797	14	3.347	1.639	-1.525	4.803	0.101
		OR, 480	2.667	-0.281	5.614	0.797	14	3.347	1.639	-1.525	4.803	0.101
		PBS	2.667	-0.281	5.614	0.797	14	3.347	1.639	-1.525	4.803	0.101
	IM, 480	OR	3.333	0.386	6.281	0.797	14	4.183	2.049	-1.360	5.457	0.019
		OR, 120	3.333	0.386	6.281	0.797	14	4.183	2.049	-1.360	5.457	0.019
		OR, 480	3.333	0.386	6.281	0.797	14	4.183	2.049	-1.360	5.457	0.019
		PBS	3.333	0.386	6.281	0.797	14	4.183	2.049	-1.360	5.457	0.019
	OR	OR, 120	-2.220×10^{-16}	-2.948	2.948	0.797	14	-2.787×10^{-16}	0.000	-2.674	2.674	1.000
		OR, 480	6.661×10^{-16}	-2.948	2.948	0.797	14	8.360×10^{-16}	8.882×10^{-16}	-2.674	2.674	1.000
		PBS	-1.776×10^{-15}	-2.948	2.948	0.797	14	-2.229×10^{-15}	-4.441×10^{-16}	-2.674	2.674	1.000
	OR, 120	OR, 480	8.882×10^{-16}	-2.948	2.948	0.797	14	1.115×10^{-15}	8.882×10^{-16}	-2.674	2.674	1.000
		PBS	-1.554×10^{-15}	-2.948	2.948	0.797	14	-1.951×10^{-15}	-4.441×10^{-16}	-2.674	2.674	1.000
	OR, 480	PBS	-2.442×10^{-15}	-2.948	2.948	0.797	14	-3.065×10^{-15}	-1.332×10^{-15}	-2.674	2.674	1.000
Day 14	IM	IM, 120	0.667	-5.302	6.635	1.613	14	0.413	0.410	-5.021	5.840	1.000
		IM, 480	-3.109×10^{-15}	-5.968	5.968	1.613	14	-1.927×10^{-15}	0.000	-5.414	5.414	1.000

Post Hoc Comparisons - Group ✻ Day - Conditional on Day

Day			Mean Difference	95% CI for Mean Difference Lower	95% CI for Mean Difference Upper	SE	df	t	Cohen's d	95% CI for Cohen's d Lower	95% CI for Cohen's d Upper	p_{bonf}
		OR	6.000	0.032	11.968	1.613	14	3.719	3.687	-2.930	10.305	0.048
		OR, 120	0.667	-5.302	6.635	1.613	14	0.413	0.410	-5.021	5.840	1.000
		OR, 480	2.000	-3.968	7.968	1.613	14	1.240	1.229	-4.331	6.790	1.000
		PBS	10.667	4.698	16.635	1.613	14	6.611	6.555	-2.109	15.220	< .001
	IM, 120	IM, 480	-0.667	-6.635	5.302	1.613	14	-0.413	-0.410	-5.840	5.021	1.000
		OR	5.333	-0.635	11.302	1.613	14	3.306	3.278	-3.106	9.661	0.109
		OR, 120	-8.882×10^{-16}	-5.968	5.968	1.613	14	-5.505×10^{-16}	0.000	-5.414	5.414	1.000
		OR, 480	1.333	-4.635	7.302	1.613	14	0.826	0.819	-4.660	6.299	1.000
		PBS	10.000	4.032	15.968	1.613	14	6.198	6.146	-2.192	14.484	< .001
	IM, 480	OR	6.000	0.032	11.968	1.613	14	3.719	3.687	-2.930	10.305	0.048
		OR, 120	0.667	-5.302	6.635	1.613	14	0.413	0.410	-5.021	5.840	1.000
		OR, 480	2.000	-3.968	7.968	1.613	14	1.240	1.229	-4.331	6.790	1.000
		PBS	10.667	4.698	16.635	1.613	14	6.611	6.555	-2.109	15.220	< .001
	OR	OR, 120	-5.333	-11.302	0.635	1.613	14	-3.306	-3.278	-9.661	3.106	0.109
		OR, 480	-4.000	-9.968	1.968	1.613	14	-2.479	-2.458	-8.437	3.520	0.557
		PBS	4.667	-1.302	10.635	1.613	14	2.892	2.868	-3.302	9.038	0.248
	OR, 120	OR, 480	1.333	-4.635	7.302	1.613	14	0.826	0.819	-4.660	6.299	1.000
		PBS	10.000	4.032	15.968	1.613	14	6.198	6.146	-2.192	14.484	< .001
	OR, 480	PBS	8.667	2.698	14.635	1.613	14	5.372	5.326	-2.388	13.041	0.002
Day 21	IM	IM, 120	0.667	-7.352	8.685	2.168	14	0.308	0.410	-6.876	7.695	1.000
		IM, 480	-1.776×10^{-15}	-8.018	8.018	2.168	14	-8.195×10^{-16}	0.000	-7.274	7.274	1.000
		OR	8.000	-0.018	16.018	2.168	14	3.691	4.917	-3.951	13.785	0.051
		OR, 120	12.667	4.648	20.685	2.168	14	5.844	7.785	-3.052	18.621	< .001
		OR, 480	6.000	-2.018	14.018	2.168	14	2.768	3.687	-4.521	11.896	0.317
		PBS	14.667	6.648	22.685	2.168	14	6.766	9.014	-2.793	20.821	< .001
	IM, 120	IM, 480	-0.667	-8.685	7.352	2.168	14	-0.308	-0.410	-7.695	6.876	1.000
		OR	7.333	-0.685	15.352	2.168	14	3.383	4.507	-4.126	13.140	0.094
		OR, 120	12.000	3.982	20.018	2.168	14	5.536	7.375	-3.152	17.902	0.002
		OR, 480	5.333	-2.685	13.352	2.168	14	2.460	3.278	-4.744	11.299	0.577
		PBS	14.000	5.982	22.018	2.168	14	6.459	8.604	-2.873	20.081	< .001
	IM, 480	OR	8.000	-0.018	16.018	2.168	14	3.691	4.917	-3.951	13.785	0.051
		OR, 120	12.667	4.648	20.685	2.168	14	5.844	7.785	-3.052	18.621	< .001
		OR, 480	6.000	-2.018	14.018	2.168	14	2.768	3.687	-4.521	11.896	0.317
		PBS	14.667	6.648	22.685	2.168	14	6.766	9.014	-2.793	20.821	< .001
	OR	OR, 120	4.667	-3.352	12.685	2.168	14	2.153	2.868	-4.984	10.720	1.000
		OR, 480	-2.000	-10.018	6.018	2.168	14	-0.923	-1.229	-8.612	6.154	1.000
		PBS	6.667	-1.352	14.685	2.168	14	3.076	4.097	-4.316	12.510	0.173
	OR, 120	OR, 480	-6.667	-14.685	1.352	2.168	14	-3.076	-4.097	-12.510	4.316	0.173
		PBS	2.000	-6.018	10.018	2.168	14	0.923	1.229	-6.154	8.612	1.000
	OR, 480	PBS	8.667	0.648	16.685	2.168	14	3.998	5.326	-3.790	14.443	0.028
Day 28	IM	IM, 120	1.333	-2.154	4.821	0.943	14	1.414	0.819	-2.455	4.094	1.000
		IM, 480	0.667	-2.821	4.154	0.943	14	0.707	0.410	-2.782	3.602	1.000
		OR	6.667	3.179	10.154	0.943	14	7.071	4.097	-1.183	9.377	< .001
		OR, 120	7.333	3.846	10.821	0.943	14	7.778	4.507	-1.118	10.131	< .001
		OR, 480	4.000	0.512	7.488	0.943	14	4.243	2.458	-1.597	6.513	0.017
		PBS	16.667	13.179	20.154	0.943	14	17.678	10.243	-0.790	21.275	< .001
	IM, 120	IM, 480	-0.667	-4.154	2.821	0.943	14	-0.707	-0.410	-3.602	2.782	1.000
		OR	5.333	1.846	8.821	0.943	14	5.657	3.278	-1.353	7.909	0.001
		OR, 120	6.000	2.512	9.488	0.943	14	6.364	3.687	-1.261	8.636	< .001
		OR, 480	2.667	-0.821	6.154	0.943	14	2.828	1.639	-1.948	5.226	0.282
		PBS	15.333	11.846	18.821	0.943	14	16.263	9.423	-0.802	19.649	< .001
	IM, 480	OR	6.000	2.512	9.488	0.943	14	6.364	3.687	-1.261	8.636	< .001
		OR, 120	6.667	3.179	10.154	0.943	14	7.071	4.097	-1.183	9.377	< .001

Post Hoc Comparisons - Group ✻ Day - Conditional on Day

Day			Mean Difference	95% CI for Mean Difference Lower	95% CI for Mean Difference Upper	SE	df	t	Cohen's d	95% CI for Cohen's d Lower	95% CI for Cohen's d Upper	p_{bonf}
		OR, 480	3.333	-0.154	6.821	0.943	14	3.536	2.049	-1.756	5.853	0.069
		PBS	16.000	12.512	19.488	0.943	14	16.971	9.833	-0.795	20.461	< .001
	OR	OR, 120	0.667	-2.821	4.154	0.943	14	0.707	0.410	-2.782	3.602	1.000
		OR, 480	-2.667	-6.154	0.821	0.943	14	-2.828	-1.639	-5.226	1.948	0.282
		PBS	10.000	6.512	13.488	0.943	14	10.607	6.146	-0.941	13.233	< .001
	OR, 120	OR, 480	-3.333	-6.821	0.154	0.943	14	-3.536	-2.049	-5.853	1.756	0.069
		PBS	9.333	5.846	12.821	0.943	14	9.899	5.736	-0.975	12.447	< .001
	OR, 480	PBS	12.667	9.179	16.154	0.943	14	13.435	7.785	-0.849	16.418	< .001

Note. P-value and confidence intervals adjusted for comparing a family of 21 estimates (confidence intervals corrected using the bonferroni method).

Generalized Linear Mixed Models-Groups

ANOVA Summary

Effect	df	ChiSq	p
Group	6	88.178	< .001

Note. Generalized linear mixed model with gaussian family and identity link function.
Note. Model terms tested with likelihood ratio tests testMethod.
Note. The following variable is used as a random effects grouping factor: 'Date'.
Note. Type III Sum of Squares

Model summary

Fit statistics

Deviance (REML)	log Lik.	df	AIC	BIC
412.198	-206.099	9	430.198	452.075

Note. The model was fitted using restricted maximum likelihood. Please note that models with different fixed effects cannot be compared when REML is used. To use ML, switch 'Test method' to 'Likelihood ratio tests'.

Sample sizes

Observations	Levels of RE grouping factors: Date
84	4

Fixed Effects Estimates

Term	Estimate	SE	t
Intercept	12.262	2.181	5.622
Group (1)	3.738	0.759	4.927
Group (2)	3.238	0.759	4.268
Group (3)	3.905	0.759	5.147
Group (4)	-1.929	0.759	-2.542
Group (5)	-1.929	0.759	-2.542
Group (6)	0.238	0.759	0.314

Note. The intercept corresponds to the (unweighted) grand mean; for each factor with k levels, k - 1 parameters are estimated with sum contrast coding. Consequently, the estimates cannot be directly mapped to factor levels. Use estimated marginal means for obtaining estimates for each factor level/design cell or their differences.

Estimated Marginal Means

Group	Estimate	SE	95% CI Lower	95% CI Upper	z	p†
IM	16.000	2.309	11.474	20.526	4.764	< .001
IM 120	15.500	2.309	10.974	20.026	4.547	< .001
IM 480	16.167	2.309	11.641	20.692	4.836	< .001
OR	10.333	2.309	5.808	14.859	2.310	0.021
OR 120	10.333	2.309	5.808	14.859	2.310	0.021
OR 480	12.500	2.309	7.974	17.026	3.248	0.001
PBS	5.000	2.309	0.474	9.526	2.885×10^{-14}	1.000

Note. Results are on the response scale.
† P-values correspond to test of null hypothesis against 5.

Generalized Linear Mixed Models-Date

ANOVA Summary

Effect	df	ChiSq	p
Date	3	84.845	< .001

Note. Generalized linear mixed model with gaussian family and identity link function.
Note. Model terms tested with likelihood ratio tests testMethod.
Note. The following variable is used as a random effects grouping factor: 'Group'.
Note. Type III Sum of Squares

Model summary

Fit statistics

Deviance (REML)	log Lik.	df	AIC	BIC
428.191	-214.096	6	440.191	454.776

Note. The model was fitted using restricted maximum likelihood. Please note that models with different fixed effects cannot be compared when REML is used. To use ML, switch 'Test method' to 'Likelihood ratio tests'.

Sample sizes

Observations	Levels of RE grouping factors: Group
84	7

Fixed Effects Estimates

Term	Estimate	SE	t
Intercept	12.262	1.543	7.949
Date (1)	-6.119	0.536	-11.406
Date (2)	0.548	0.536	1.021
Date (3)	1.405	0.536	2.618

Note. The intercept corresponds to the (unweighted) grand mean; for each factor with k levels, k - 1 parameters are estimated with sum contrast coding. Consequently, the estimates cannot be directly mapped to factor levels. Use estimated marginal means for obtaining estimates for each factor level/design cell or their differences.

Estimated Marginal Means

Date	Estimate	SE	95% CI Lower	95% CI Upper	z	p†
7	6.143	1.633	2.942	9.344	3.761	< .001
14	12.810	1.633	9.609	16.010	7.843	< .001
21	13.667	1.633	10.466	16.868	8.368	< .001

Estimated Marginal Means

			95% CI			
Date	Estimate	SE	Lower	Upper	z	p†
28	16.429	1.633	13.228	19.630	10.059	< .001

Note. Results are on the response scale.
† P-values correspond to test of null hypothesis against 0.
