# Supplementary material for: Utilizing NF-κB Signaling in Porcine Epithelial Cells to Identify a Plant-Based Additive for the Development of a Porcine Epidemic Diarrhea Virus Vaccine
Source: Vet Sci. 2025 Feb 18;12(2):181. doi: 10.3390/vetsci12020181 (PMC11860592; doi:10.3390/vetsci12020181)
Supplement: Supplementary file 1 [file vetsci-12-00181-s001.zip › Supplementary File S4.pdf]

Supplementary File S4

Supplemental Data Figure 8

Results

ANOVA

ANOVA - IgA titer

Homogeneity Correction	Cases	Sum of Squares	df	Mean Square	F	p	ω²	95% CI for ω² Lower	95% CI for ω² Upper
None	Group	25.143	6.000	4.190	3.259	0.032	0.392	0.000	0.600
	Residuals	18.000	14.000	1.286					
Welch	Group	25.143	6.000	4.190	1.065	0.469	0.392	0.000	0.600
	Residuals	18.000	6.197	2.905					

Note. Type III Sum of Squares

Descriptives

Descriptives - IgA titer

Group	N	Mean	SD	SE	Coefficient of variation
IM	3	1.667	0.577	0.333	0.346
IM 120	3	1.667	0.577	0.333	0.346
IM 480	3	2.333	0.577	0.333	0.247
OR	3	2.333	0.577	0.333	0.247
OR 120	3	2.333	0.577	0.333	0.247
OR 480	3	5.000	2.646	1.528	0.529
PBS	3	1.667	0.577	0.333	0.346

Assumption Checks

Test for Equality of Variances (Levene's)

F	df1	df2	p
5.939	6.000	14.000	0.003

Post Hoc Tests

Standard (HSD)

Post Hoc Comparisons - Group

		Mean Difference	SE	df	t	p_{tukey}
IM	IM 120	-9.992×10^{-16}	0.926	14	-1.079×10^{-15}	1.000

Post Hoc Comparisons - Group

		Mean Difference	SE	df	t	p_{tukey}
	IM 480	-0.667	0.926	14	-0.720	0.989
	OR	-0.667	0.926	14	-0.720	0.989
	OR 120	-0.667	0.926	14	-0.720	0.989
	OR 480	-3.333	0.926	14	-3.600	0.036
	PBS	5.551×10^{-17}	0.926	14	5.996×10^{-17}	1.000
IM 120	IM 480	-0.667	0.926	14	-0.720	0.989
	OR	-0.667	0.926	14	-0.720	0.989
	OR 120	-0.667	0.926	14	-0.720	0.989
	OR 480	-3.333	0.926	14	-3.600	0.036
	PBS	1.055×10^{-15}	0.926	14	1.139×10^{-15}	1.000
IM 480	OR	1.041×10^{-15}	0.926	14	1.124×10^{-15}	1.000
	OR 120	5.690×10^{-16}	0.926	14	6.146×10^{-16}	1.000
	OR 480	-2.667	0.926	14	-2.880	0.126
	PBS	0.667	0.926	14	0.720	0.989
OR	OR 120	-4.718×10^{-16}	0.926	14	-5.097×10^{-16}	1.000
	OR 480	-2.667	0.926	14	-2.880	0.126
	PBS	0.667	0.926	14	0.720	0.989
OR 120	OR 480	-2.667	0.926	14	-2.880	0.126
	PBS	0.667	0.926	14	0.720	0.989
OR 480	PBS	3.333	0.926	14	3.600	0.036

Note. P-value adjusted for comparing a family of 7 estimates.

Kruskal-Wallis Test

Kruskal-Wallis Test

					95% CI for Rank ε²	
Facto r	Statistic	df	p	Rank ε²	Lower	Upper
Group	12.032	6	0.061	0.602	0.602	0.876

Dunn

Dunn's Post Hoc Comparisons - Group

Comparison	z	W_i	W_j	r_{rb}	p	p_{bonf}	p_{holm}
IM - IM 120	0.000	7.000	7.000	0.000	1.000	1.000	1.000
IM - IM 480	-1.137	7.000	12.167	0.556	0.256	1.000	1.000
IM - OR	-1.137	7.000	12.167	0.556	0.256	1.000	1.000
IM - OR 120	-1.137	7.000	12.167	0.556	0.256	1.000	1.000
IM - OR 480	-2.750	7.000	19.500	1.000	0.006	0.125	0.125
IM - PBS	0.000	7.000	7.000	0.000	1.000	1.000	1.000
IM 120 - IM 480	-1.137	7.000	12.167	0.556	0.256	1.000	1.000
IM 120 - OR	-1.137	7.000	12.167	0.556	0.256	1.000	1.000
IM 120 - OR 120	-1.137	7.000	12.167	0.556	0.256	1.000	1.000
IM 120 - OR 480	-2.750	7.000	19.500	1.000	0.006	0.125	0.125
IM 120 - PBS	0.000	7.000	7.000	0.000	1.000	1.000	1.000
IM 480 - OR	0.000	12.167	12.167	0.000	1.000	1.000	1.000

Dunn's Post Hoc Comparisons - Group

Comparison	z	W_i	W_j	r_{rb}	p	p_{bonf}	p_{holm}
IM 480 - OR 120	0.000	12.167	12.167	0.000	1.000	1.000	1.000
IM 480 - OR 480	-1.613	12.167	19.500	0.889	0.107	1.000	1.000
IM 480 - PBS	1.137	12.167	7.000	0.556	0.256	1.000	1.000
OR - OR 120	0.000	12.167	12.167	0.000	1.000	1.000	1.000
OR - OR 480	-1.613	12.167	19.500	0.889	0.107	1.000	1.000
OR - PBS	1.137	12.167	7.000	0.556	0.256	1.000	1.000
OR 120 - OR 480	-1.613	12.167	19.500	0.889	0.107	1.000	1.000
OR 120 - PBS	1.137	12.167	7.000	0.556	0.256	1.000	1.000
OR 480 - PBS	2.750	19.500	7.000	1.000	0.006	0.125	0.125

Note. Rank-biserial correlation based on individual Mann-Whitney tests.
